# Molecular Detection and Genetic Characterization of Novel RNA Viruses in Wild and Synanthropic Rodents and Shrews in Kenya

**DOI:** 10.3389/fmicb.2019.02696

**Published:** 2019-11-21

**Authors:** Samson Omondi Onyuok, Ben Hu, Bei Li, Yi Fan, Kelvin Kering, Griphin Ochieng Ochola, Xiao-Shuang Zheng, Vincent Obanda, Sheila Ommeh, Xing-Lou Yang, Bernard Agwanda, Zheng-Li Shi

**Affiliations:** ^1^CAS Key Laboratory of Special Pathogens and Biosafety, Wuhan Institute of Virology, Chinese Academy of Sciences, Wuhan, China; ^2^Sino-Africa Joint Research Center, Chinese Academy of Sciences, Wuhan, China; ^3^Mammalogy Section, National Museums of Kenya, Nairobi, Kenya; ^4^University of Chinese Academy of Sciences, Beijing, China; ^5^Veterinary Services Department, Kenya Wildlife Service, Nairobi, Kenya; ^6^Institute of Biotechnology Research, Jomo Kenyatta University of Science and Technology, Nairobi, Kenya

**Keywords:** Kenya, RNA viruses, zoonotic pathogens, rodents, shrews, arenavirus

## Abstract

The majority of emerging and reemerging zoonotic viral pathogens are RNA viruses. Pathogen discovery programs of emerging infectious diseases (EIDs) in wildlife have implicated rodents and shrews as hosts of diverse human pathogens, such as hantaviruses, arenaviruses, paramyxoviruses, etc. Despite these threats, little is known about the diversity of viruses circulating among rodents and shrews in Kenya, meaning the risk of infectious disease outbreak from these small mammals could be oblivious. This study reports the first surveillance toward understanding the diversity of RNA viruses carried by rodents and shrews in areas of high-potential contact with humans in Kenya through molecular detection. A total of 617 samples comprising fecal, urine, and tissues from 138 rodents and 5 shrews were screened for eight different families of viruses using RT-PCR assays. The results highlight the presence of diverse astroviruses, paramyxoviruses, hepeviruses, and arenavirus, circulating in both wild and synanthropic Kenyan rodents and shrews. Most of the viruses detected in this study are novel strains and some belong to the families that contain important human viral pathogens. Notably, a novel arenavirus was detected in *Grammomys macmillani*, a rodent species newly identified to harbor arenavirus, and it potentially represent a novel arenavirus species. Our findings demonstrate the need for continued pathogen surveillance among these small mammals as well as among the vulnerable and exposed livestock and humans. This would help in development and implementation of effective preventive and control strategies on EIDs in countries with rich wildlife biodiversity like Kenya.

## Introduction

More than half of the documented human pathogens are zoonotic, and emerging infectious diseases (EIDs) are more likely to be caused by pathogens of zoonotic origin (Woolhouse and Gowtage-Sequeria, 2005; Jones et al., 2008). In most instances, high impact outbreaks of the past decades and present pandemics have resulted from pathogens of wildlife origin, which usually cause little or no clinical signs of infection in their natural hosts (Wu et al., 2018), but with devastating global health and economic effects when they spill over into humans and livestock (Allen et al., 2017). The present global upsurge in outbreaks of emerging and re-emerging infectious diseases has necessitated the search for reservoirs of zoonoses of public health importance in order to develop resilient response strategies.

RNA viruses account for a large part of the known zoonotic pathogens owing to their often high rates of mutation and capacity to infect and adapt in a wide range of hosts (Woolhouse and Gowtage-Sequeria, 2005; Jones et al., 2008; Meheretu et al., 2012). Surveillance and discovery programs of EIDs in wildlife have identified rodents and shrews as natural reservoirs of diverse RNA viruses such as hantaviruses, arenaviruses, astroviruses, picornaviruses, paramyxoviruses, etc. (Klempa et al., 2006; Meheretu et al., 2012; Firth et al., 2014; Hu et al., 2014; Sasaki et al., 2014; Gryseels et al., 2015; Gouy de Bellocq et al., 2016; Těšíková et al., 2017; Wang et al., 2017). It is believed that these viruses establish themselves in their hosts through co-evolution and are maintained in nature by both vertical and horizontal transmissions (Streicker, 2013). Spillover events into humans and livestock result from encounters with these small mammals whose presence around human dwellings is on the rise either as pests or as food (Bonwitt et al., 2016). Evidently this poses a potential threat to public health and it is imperative that pathogen surveillance and discovery programs are implemented on high risk wildlife groups including rodents and shrews.

Kenya is a country rich in wildlife diversity, with a nationwide distribution of about 106 rodent and 36 shrew species (Musila et al., 2019). Expansion in agricultural activities among other anthropogenic factors encourages encroachment into wildlife habitats and amplifies the frequency of human contacts with these animals and pathogens carried by them (Young et al., 2017). However, there is currently no documentation of viruses harbored by rodents and shrews in Kenya yet they have been sighted and trapped around human dwellings. Also, there could be a possibility of misdiagnosis and underreporting of human infections with rodent-borne viruses in the country. These gaps justify the need to investigate and characterize the diversity of viruses in rodents and shrews and quantify the possible impact of these viruses on public health in Kenya. It was against this backdrop that we carried out this study, in which both wild and peridomestic species of these animals around and within agro-ecological zones near human habitations were sampled and screened for a variety of RNA viral families. This work thus constitutes the first virus surveillance study in Kenyan rodents and shrews, and provides baseline data for understanding the distribution, genetic diversity, as well as potential spillover risk of RNA viruses circulating in small mammals in Kenya.

## Materials and Methods

### Study Area and Sample Collection

**FIGURE 1:**
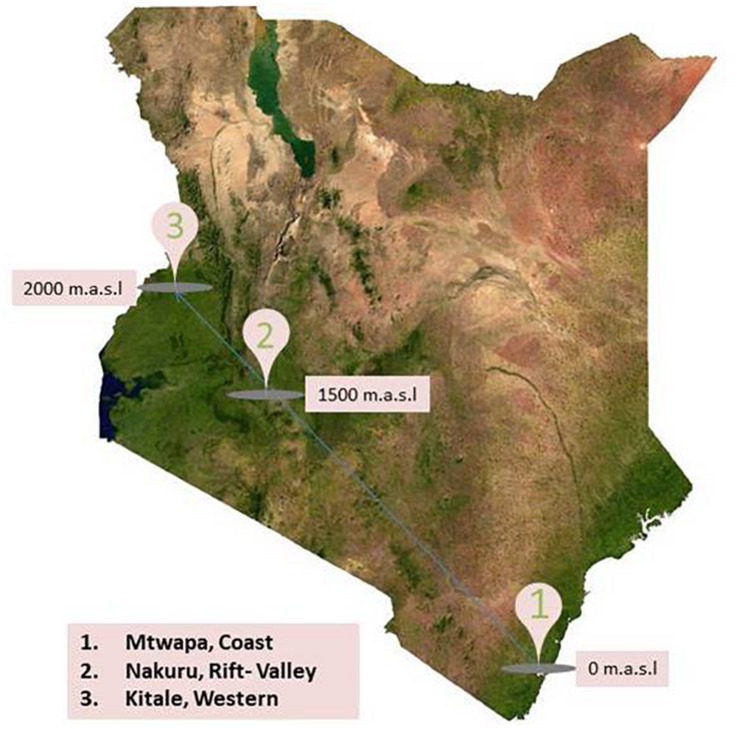
Map of Kenya showing rodent and shrew sampling sites between August and September, 2016.

Field component of this research was conducted in Kenya, a country rich in wildlife, with a varied climate ranging from the tropical climate in the coastal region, hot dry lowlands, and temperate highlands. Three specific locations were selected for this study: (a) Mtwapa, in the Coast at 0 m above sea level (m.a.s.l), (b) Nakuru, in the Rift Valley at 1500 m.a.s.l., and (c) Kitale, in Western at 2000 m.a.s.l. (Figure 1). Each location provides unique ecotype in a high human density neighborhood with intensive large and small scale farming practices indicating high probability of human contacts with rodents and shrews. Line transects were established in homes (compound, kitchens, and stores), farms, and natural habitats (bushes and forests) between August and September, 2016. In every transect, a combination of peanut butter with oat meal as well as fish was used as bait in 20–50 Sherman and Museum Special traps.

Captured animals were sedated via intramuscular injection with ketamine and knocked off by cervical dislocation. Individuals were taxonomically identified using the field guide (Kingdom et al., 2013), and their morphometrics were taken. The animals were necropsied and the following tissues were aseptically collected in cryotubes: lung, liver, kidney, and spleen. Fecal pellets and urine were collected only when available, in both RNALater and VTM. All samples were kept in liquid nitrogen and shipped to Wuhan Institute of Virology, China, for storage at −80°C and for analysis.

### Molecular Detection of Viruses and Phylogenetic Analysis

RNA was extracted using a viral RNA extraction kit (Roche, Germany) according to the manufacturer’s instructions. The first strand cDNA was synthesized with superscript III reverse transcriptase (Invitrogen) and random hexamers. PCR assays utilizing the Platinum Taq DNA polymerase kit (Invitrogen) were applied to screen the presence of following RNA viruses: hantaviruses (Klempa et al., 2006), arenaviruses (Li et al., 2015), coronaviruses (Watanabe et al., 2010), paramyxoviruses (Tong et al., 2008), astroviruses (Chu et al., 2008), hepeviruses (Drexler et al., 2012), picornaviruses (Lau et al., 2011), and flaviviruses (Moureau et al., 2007). The information of primers used in PCR testing of different viral families are presented in Table 1. Hantaviruses, arenaviruses, and flaviviruses were screened using kidney, liver, lung, and spleen tissues. Feces were tested for astroviruses, coronaviruses, paramyxoviruses, and picornaviruses which are known to infect gastrointestinal tract. Liver tissues were tested for hepeviruses as liver is the major target tissue for hepatitis E virus infection. Since paramyxoviruses can be secreted in urine, we also screened kidney and the nine urine samples for paramyxovirus RNA. Identities of positive host species were confirmed by sequencing of mitochondrial *Cyt b* gene, recommended for species identification (Bowen et al., 1997). Nucleotide sequences of products with expected amplicon sizes were determined by Sanger sequencing. Sequences were aligned with representative members of relevant family using ClustalW. Phylogenetic inference of each alignment was done in MEGA version 7.0, using the maximum-likelihood (ML) approach and the Tamura–Nei model with a bootstrap of 1000 replicates (Hall, 2013).

**TABLE 1:** Sequences of primers used for RT-PCR screening.

**Virus**	**Primer name**	**Primer sequence (5′–3′)**	**Region**	**Amplicon size**
Arenavirus	Arena-F1	AYNGGNACNCCRTTNGC	L gene	Round 1 938 bp
	Arena-R1	TCHTAYAARGARCARGTDGGDGG		
	Arena-F2	GGNACYTCHTCHCCCCANAC		Round 2 610 bp
	Arena-R2	AGYAARTGGGGNCCNAYKATG		
Astrovirus	AstroFWD1	GARTTYGATTGGRCKCGKTAYGA	RdRp	Round 1 436 bp
	AstroFWD2	GARTTYGATTGGRCKAGGTAYGA		
	AstroRVS1	GGYTTKACCCACATNCCRAA		
	AstroFWD3	CGKTAYGATGGKACKATHCC		Round 2 421 bp
	AstroFWD4	AGGTAYGATGGKACKATHCC		
Coronavirus	CoV-FWD3	GGTTGGGAYTAYCCHAARTGTGA	RdRp	Round 1 440 bp
	CoV-RVS3	CCATCATCASWYRAATCATCATA		
	CoV-FWD4	GAYTAYCCHAARTGTGAUMGWGC		Round 2 434 bp
Flavirirus	Flavi-FWD	TGYRBTTAYAACATGATGGG	NS5 gene	270 bp
	Flavi-RVS	GTGTCCCAICCNGCNGTRTC		
Hantavirus	HAN-L-F1	ATGTAYGTBAGTGCWGATGC	L gene	Round 1 453 bp
	HAN-L-R1	AACCADTCWGTYCCRTCATC		
	HAN-L-F2	TGCWGATGCHACIAARTGGTC		Round 2 385 bp
	HAN-L-R2	GCRTCRTCWGARTGRTGDGCAA		
Hepevirus	DE-F4228	ACYTTYTGTGCYYTITTTGGTCCITGGTT	RdRp	Round 1 371 bp
	DE-R4598	CCGGGTTCRCCIGAGTGTTTCTTCCA		
	DE-R4565	GCCATGTTCCAGAYGGTGTTCCA		Round 2 338 bp
Paramyxovirus	PAR-F1	GAAGGITATTGTCAIAARNTNTGGAC	*pol* gene	Round 1 639 bp
	PAR-R	GCTGAAGTTACIGGITCICCDATRTTNC		
	PAR-F2	GTTGCTTCAATGGTTCARGGNGAYAA		Round 2 561 bp
Picornavirus	Picorna-F	CYTATHTRAARGATGAGCTKAGA	3D^pol^	571 bp
	Picorna-R	GCAATNACRTCATCKCCRTA		

### Genomic Characterization of the Novel Arenavirus

Isolation of arenavirus was attempted with spleen sample. Vero cells were inoculated with the spleen homogenate and observed daily for cytopathic effect. For next-generation sequencing (NGS), library was constructed using Illumina Truseq Stranded mRNA Sample Preparation Kit (Illumina, San Diego, CA, United States) following the manufacturer’s instructions and sequencing was performed on an HiSeq 3000 sequencer (Illumina, San Diego, CA, United States). *De novo* assembly of NGS data was performed using Trinity version r2011-08-20 (Grabherr et al., 2011) and the resulting contigs were aligned to the non-redundant nucleotide database on NCBI. Contigs mapping significantly to the reference sequences were then retrieved and re-mapped to the full genome reference to generate a consensus sequence. The genome end sequence was amplified through the SMARTer^®^ RACE 5′/3′ Kit (Takara). The recovered genome sequence was used for ORF prediction and aligned with those of representative members of mammarenavirus using ClustalW. Pairwise sequence similarities of the L/S segments and ORFs of the novel arenavirus to other mammarenaviruses were calculated using DNAstar. Phylogenetic inference of the aligned homologs of the four ORFs was done in MEGA version 7.0, using the ML approach and the Tamura–Nei model with a bootstrap of 1000 replicates.

## Results

### Rodents and Shrew Samples

A total of 617 tissue and fecal samples from 143 individual small mammals of 18 species (17 rodent species and 1 shrew species) were collected between August and September, 2016 from the three distinct sampling localities in Kenya (Mtwapa, Nakuru, and Kitale). The samples comprised 138 lungs, 142 livers, 138 kidneys, 134 spleens, 56 fecal samples, and 9 urine samples (Table 2).

**TABLE 2:** Numbers of individual animals and numbers of each type of sample per species collected in this study.

**Species**	**Number of individual animals**				**Number of each type of sample**					
	**Mtwapa**	**Nakuru**	**Kitale**	**Total**	**Kidney**	**Liver**	**Spleen**	**Lung**	**Feces**	**Urine**
*Aethomys kaiseri*	0	10	0	10	10	10	10	10	4	1
*Arvicanthis niloticus*	0	7	4	11	11	11	11	11	5	–
*Aterelix albiventrix*	1	0	0	1	1	1	1	1	1	1
*Cricetomys gambianus*	0	1	0	1	1	1	1	1	1	1
*Crocidura Olivieri*	0	3	2	5	4	5	5	4	–	–
*Gerbilliscus robustus*	5	0	0	5	4	5	4	4	3	–
*Grammomys macmillani*	0	0	2	2	2	2	2	2	1	–
*Graphiurus murinus*	0	0	2	2	2	2	2	2	2	1
*Lemniscomys striatus*	0	5	2	7	7	7	5	7	–	–
*Lophuromys aquilus*	0	0	6	6	6	6	6	6	5	3
*Mastomys natalensis*	0	37	14	51	50	51	50	50	17	1
*Mus minutoides*	0	10	2	12	11	11	9	11	3	–
*Mus triton*	0	5	4	9	9	9	7	9	1	–
*Oenomys hypoxanthus*	0	0	1	1	1	1	1	1	1	–
*Otomys tropicalis*	0	1	0	1	1	1	1	1	–	–
*Paraxerus ochraceus*	2	0	0	2	2	2	2	2	–	–
*Rattus rattus*	2	3	10	15	14	15	14	14	10	1
*Tachyoryctes splendens*	0	2	0	2	2	2	2	2	2	–
Total	10	84	49	143	138	142	134	138	56	9

### Virus Detection

Viruses of four families, astroviruses, paramyxoviruses, hepevirus, and arenaviruses, were detected in 17 out of 143 animals, among which eight were from Nakuru and nine were from Kitale. The positive rates ranged from 0.7 (1/142, arenavirus) to 12.5% (7/56, astrovirus) (Table 3). However, none of these small mammals tested positive for hantavirus, coronavirus, picornavirus, or flavivirus. There was no detection of viruses from any of the samples from Mtwapa, possibly due to a biased small sample size (Tables 2, 3).

**TABLE 3:** Individual animals and sample types positive for four viral families.

		**Number of positives/number**				**Number of positives/number of tested samples**					
		**of tested individuals**									
**Virus**	**Positive species**	**MT**	**NK**	**KT**	**Total**	**Feces**	**Lung**	**Liver**	**Kidney**	**Spleen**	**Urine**
Astrovirus	*C. gambianus*	–	1/1	–	1/1	1/1	–	–	–	–	–
	*M. natalensis*	–	4/7	0/10	4/17	4/17	–	–	–	–	–
	*R. rattus*	0/1	0/2	2/7	2/10	2/10	–	–	–	–	–
Paramyxovirus	*L. aquilus*	–	–	2/6	2/6	0/5	–	–	1/6	–	1/3
	*M. natalensis*	–	0/37	1/14	1/51	0/17	–	–	1/50	–	0/1
	*M. minutoides*	–	1/10	0/2	1/12	0/3	–	–	1/11	–	–
	*M. triton*	–	2/5	1/4	3/9	0/1	–	–	3/9	–	–
Hepevirus	*C. olivieri*	–	0/3	1/2	1/5	–	–	1/5	–	–	–
	*R. rattus*	0/2	0/3	1/10	1/15	–	–	1/15	–	–	–
Arenavirus	*G. macmillani*	–	–	1/2	1/2	–	1/2	1/2	1/2	1/2	–

### Astrovirus

**FIGURE 2:**
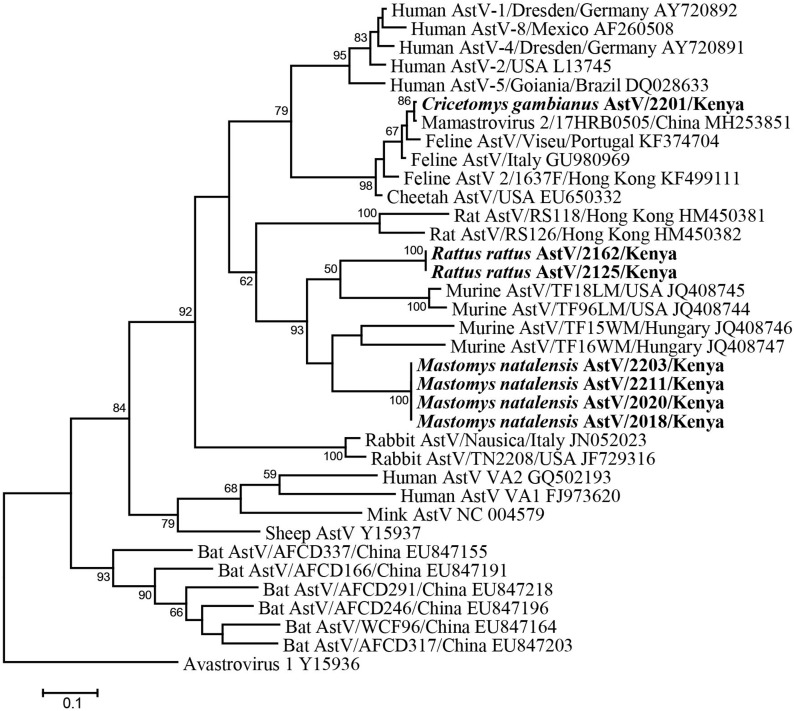
ML tree based on 315-nt partial RdRp gene of astroviruses (corresponding to human astrovirus 1 genome nt 3619-3933, AY720892). Strains detected in this study are indicated in bold.

Fifty-six fecal samples were screened for astroviruses by nested PCR targeting the RNA-dependent RNA polymerase (RdRp) gene of all known mammalian astroviruses. Astroviruses were detected in seven rodents from three species (Table 3). The prevalence rate in *Mastomys natalensis* and *Rattus rattus* was 23.5 (4/17) and 20.0% (2/10), respectively. The only one *Cricetomys gambianus* sampled in this study was also found to harbor astrovirus. Phylogenetic tree of the partial RdRp gene showed that astroviruses detected in Kenyan rodents could be divided into three host-specific groups (Figure 2). The *R. rattus* astroviruses and *M. natalensis* astroviruses were clustered with previously reported rodent astroviruses, but these two novel clades from Kenya were distinct from the existing strains, sharing lower than 86% nucleotide sequence identity. The single astrovirus detected in *C. gambianus* was exceptionally interesting, as it was closely related to feline astroviruses, sharing 93–99% nt sequence identities to strains from domestic cats and cheetah (Figure 2), which implied the possibility of cross-species transmission between rodents and cats.

### Paramyxovirus

A total of 138 kidney samples, 56 fecal samples, and 9 urine samples were tested for paramyxovirus RNA. Seven samples tested positive, including six kidney tissues and one urine from four rodent species (Table 3). Among the six individuals of *Lophuromys aquilus*, paramyxovirus was discovered in two animals (33.3%), with one positive in kidney and the other positive in urine. *Mus triton* had an equivalent prevalence rate, in which three out of nine animals were positive (Table 3).

**FIGURE 3:**
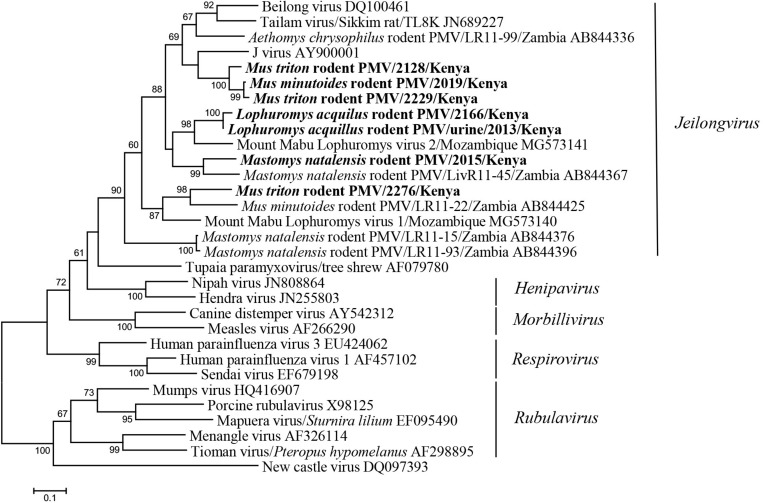
ML tree based on 506-nt partial L gene of paramyxoviruses (corresponding to Beilong virus genome nt 14889-15394, DQ100461). Strains detected in this study are indicated in bold.

The seven newly identified Kenyan rodent paramyxoviruses showed <82% nt sequence identities to known paramyxoviruses. In the phylogenetic tree, they fell into the large clade of the newly recognized genus *Jeilongvirus*. The Kenyan rodent paramyxoviruses were genetically diverse and formed four distinct clusters, most of which exhibited host species specificity (Figure 3). Three strains from *M. triton* and *Mus minutoides* were phylogenetically related to J virus. The paramyxoviruses discovered in *M. natalensis* and *L. aquilus* from Kenya were most closely related to strains previously found in these two species, respectively, from other African countries (Figure 3).

### Hepevirus

**FIGURE 4:**
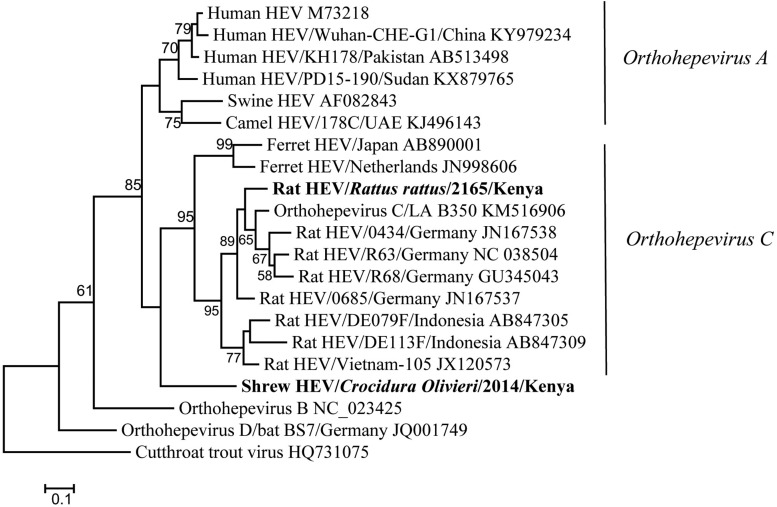
ML tree based on 254-nt partial RdRp gene of hepeviruses (corresponding to Orthohepevirus C strain LA-B350 genome nt 4145-4398, KM516906). Strains detected in this study are indicated in bold.

Hemi-nested RT-PCR was applied to screen a total of 142 liver tissues, and hepevirus RNA was found in two specimens. One positive sample originated from *R. rattus*, with the detection rate of 6.67% (1/15) (Table 3). The Kenyan rat hepevirus shared <87% nt sequence identity with rat hepeviruses reported in Europe and Asia in the partial RdRp gene. It was placed phylogenetically in the clade consisting of rodent-specific hepeviruses, and can be identified as a novel variant within the *Orthohepevirus C* species (Figure 4).

In addition to rodents, novel shrew hepevirus sequence was detected in one of the five samples from *Crocidura olivieri* (Table 3). The amino acid sequence identities of partial RdRp between the shrew hepevirus and known hepeviruses were no >76%. Phylogenetic analysis revealed that the shrew hepevirus was divergent from rodent hepevirus and formed a separate monophyletic branch (Figure 4).

### Genomic Characterization of a Novel Arenavirus

Kidney, liver, lung, and spleen tissues from 143 rodent and shrews were screened for presence of arenavirus RNA by RT-PCR targeting partial L gene. One out of the two *Grammomys macmillani* individuals was arenavirus PCR positive, and the virus was detected in all four types of tissue (Table 3). The animal infected with arenavirus was trapped in a bush on the outskirts of Kitale town in western Kenya. The newly identified arenavirus was thus designated as Kitale virus under the name of its geographical origin.

The viral RNA extracted from the positive spleen sample was used for NGS to determine genome sequence of Kitale virus. Assembly allowed reconstruction of complete coding sequences of its S and L segments. Each of the two segments of Kitale virus genome has the typical two open-reading frames (ORFs) in ambisense orientation, separated by the stem–loop structures. The S segment contains genes for glycoprotein precursor (GPC, 493 aa) and nucleoprotein (NP, 566 aa), while the L segment encodes the RdRp, also known as the L protein (2225 aa), and matrix Z protein (96 aa). In the L ORF of Kitale virus, the endonuclease motif (P_88_, D_89_, E_102_, and K_115_) is well conserved like other arenaviruses (Vieth et al., 2004). The two late domains PTAP and PPPY are also contained in the Z protein as in most arenaviruses including Lassa virus (LASV).

We compared the nucleotide and deduced amino acid (aa) sequences of the four ORFs of Kitale virus and other representative Old-World arenaviruses (OWA) (Table 4). In both of the two genomic segments and all four genes, Kitale virus shared the highest sequence identity with Ippy virus, which infected *Arvicanthis* in Central Africa Republic. The nucleotide/aa sequence identities between Kitale virus and Ippy virus were 71.4/80.5 and 69.5/78.1% for GPC and NP genes, respectively. Sequence identities in the L segment were substantially lower, with 61.5/62.2% (L) and 55.7/61.1% (Z). None of the other known OWAs has >60% sequence identity with Kitale virus in the L protein (Table 4). Furthermore, analysis using the PAirwise Sequence Comparison (PASC) tool revealed that the closest hits for both S and L segments of Kitale virus were Ippy virus. The pairwise sequence identities between Kitale virus and Ippy virus were 69.27 and 60% for S and L segments, respectively, which are below the cut-off values (80 and 76%) recommended by the International Committee on Taxonomy of Viruses (ICTV) for arenavirus species demarcation, justifying the recognition of Kitale virus as a new species in the genus *Mammarenavirus* (Radoshitzky et al., 2015; Blasdell et al., 2016).

**TABLE 4:** Sequence comparison of the four ORFs of Kitale virus and selected mammarenaviruses.

**Mammarenavirus species^∗^**	**nt/aa sequence identities**			
	**Z**	**L**	**GPC**	**NP**
Ippy virus	55.7/61.1	61.5/62.2	71.4/80.5	69.5/78.1
Mariental virus	50.2/55.9	57.5/52.6	66.8/73.6	67.6/73.5
Loie River virus	54.7/56.5	57.0/56.3	65.2/69.5	66.4/71.1
Lassa virus	54.3/57.0	58.5/55.3	67.0/72.4	65.1/70.6
Wenzhou virus	53.0/51.6	57.1/55.1	63.0/67.2	67.3/73.7
Luna virus	57.7/57.6	56.7/54.3	69.0/73.7	64.7/68.0
Merino Walk virus	48.5/51.7	56.0/53.6	62.3/68.3	64.8/67.6
Gairo virus	63.1/54.8	57.2/54.7	66.8/71.1	64.4/69.9
Mopeia virus	55.7/57.9	56.8/54.7	66.6/71.7	65.9/69.6
LCMV	47.3/50.0	51.9/47.3	61.8/61.2	62.3/63.8
Lujo virus	46.1/47.2	50.4/43.5	52.8/42.3	59.3/58.4
Mobala virus	56.5/60.0	56.8/54.9	67.5/72.0	65.4/69.8

*^∗^The GenBank accession numbers of the reference mammarenavirus genome sequences are as following: Ippy virus (NC_007905, NC_007906), Mariental virus (NC_027134, NC_027136), Loie River virus (KC669692, KC669697), Lassa virus (KF478762, KF478765), Wenzhou virus (NC_026018, NC_026019), Luna virus (NC_016152, NC_016153), Merino Walk virus (NC_023763, NC_023764), Gairo virus (NC_026246, NC_026247), Mopeia virus (DQ328874, DQ328875), LCMV (FJ607025, FJ607036), Lujo virus (NC_012776, NC_012777), and Mobala virus (NC_007903, NC_007904).*

**FIGURE 5:**
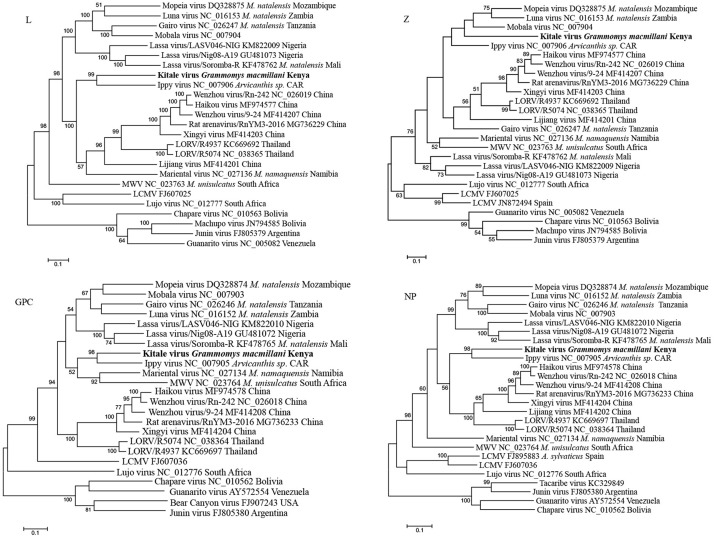
Phylogenetic analysis of complete L, Z, GPC, and NP genes of mammarenaviruses. The novel *Grammomys macmillani* arenavirus identified in this study is indicated in bold. MWV, Merino Walk virus; LORV, Loie River virus; LCMV, Lymphocytic choriomeningitis virus. *M. natalensis*, *Mastomys natalensis*; *M. namaquensis*, *Micaelamys namaquensis*; *M. unisulcatus*, *Myotomys unisulcatus*. CAR, Central African Republic.

Phylogenetic trees from the entire L, GCP, and NP genes placed Kitale virus with Ippy virus in a monophyletic clade (Figure 5). The phylogeny of GPC gene revealed that African arenaviruses, including Kitale virus, were divergent from Asian arenaviruses. However, in the phylogenetic tree of the L gene, Kitale virus and Ippy virus were more related to the cluster of Wenzhou-like viruses found in Asia than to other African arenaviruses (Figure 5).

## Discussion

Rodents, bats, and shrews constitute the three largest groups of mammalian species. They have been characterized as reservoir hosts of a variety of viruses including emerging viruses that cause human diseases (Meerburg et al., 2009; Shi, 2013). East Africa is a tropical region inhabited by rich diversity of these small mammals. A large number of viruses from various families were detected in Kenyan bats in a country-wide bat virus surveillance (Waruhiu et al., 2017). Novel rodent viruses have also been described from Tanzania and Ethiopia (Meheretu et al., 2012; Gryseels et al., 2015). However, little is known about diversity and spillover potential of the viruses carried by rodents in Kenya. We hereby report the first virus surveillance research targeting Kenyan rodent and shrew populations. In our study, novel RNA viruses belonging to four families, *Astroviridae*, *Paramyxoviridae*, *Hepeviridae*, and *Arenaviridae*, have been found circulating among rodent and shrew communities within areas of intensive agricultural activities and human settlements in Kenya.

Astroviruses, which cause mild to severe gastroenteritis in humans and animals, infect a broad range of host species, and thus pose a potential zoonotic risk for human populations especially in regions with poor hygiene condition. In recent years, an increasing number of astroviruses have been discovered in urban and wild rodents globally (Chu et al., 2010; Farkas et al., 2012; Hu et al., 2014). Although with large diversity, the phylogeny of mammalian astroviruses shows grouping with host restriction, in which most of rodent astroviruses fall into one big clade and are further clustered by rat, mice, and vole host species (Mombo et al., 2019). In this study, we detected two different novel astroviruses in fecal samples from Kenya corresponding to two different species *R. rattus* and *M. natalensis*, which tend to be species specific. They are clustered within the murine astroviruses, but more information about the full-length capsid gene is required to determine whether they can be considered novel genotype or species.

Interestingly, this study describes for the first time a rodent infected by feline astrovirus. This finding indicates the potential cross-species infection of astroviruses between cats and rodents. Feline astrovirus infects felids including domestic cats and cheetahs, and has been worldwide reported (Atkins et al., 2009). However, viruses detected in different geographical locations and in different felid species are closely related, suggesting that feline astrovirus has existed and evolved in cats for long history. Hence, the infection of feline astrovirus in *C. gamnianus* from Kenya was more likely to result from the cross-species transmission from cats to rodents. The *C. gambianus* individual in this study was captured in a farm store where this rodent has a high likelihood of interactions with domestic cats. However, as the virus was detected in feces of the rodent, there also existed the possibility that the *C. gambianus* ingested foods contaminated by domestic cats. The virus passed intact through its gastrointestinal tract, but did not actually infect the rodent.

In the past decades, a growing number of new paramyxoviruses have been discovered from rodents. These include J-virus from mice (Jack et al., 2005), Beilong virus from rat-derived cell line (Li et al., 2006), Tailam virus from Sikkim rats (Woo et al., 2011), Mossman virus from wild rats in Australia (Miller et al., 2003), Nariva virus from the Trinidadian rat (Lambeth et al., 2009), and groups of novel paramyxoviruses reported more recently from various rodent species in Africa (Sasaki et al., 2014; Vanmechelen et al., 2018). Owing to their similar rodent hosts and close phylogenetic relationship, according to the latest ICTV taxonomy release, these viruses compose a new paramyxovirus genus named *Jeilongvirus* (Rima et al., 2018). In this study, we present diverse novel paramyxoviruses found in rodents from Kenya, all of which cluster within the genus *Jeilongvirus*. Within these large clade, the phylogenetic clustering of most Kenyan paramyxoviruses showed host specificity, as different viruses are grouped separately with those from the same rodent species or genus described in other African countries. For example, two viruses from *L. aquilus* constitute a lineage with another *Lophuromys* paramyxovirus from Mozambique. Five viruses from four rodent species in Kenya detected in our study showed distinction from each other, as well as from previously reported ones, further expanding the genetic diversity of jeilongviruses. The results provide additional support for recognition of rodents as the important natural reservoir of this paramyxovirus genus.

Members of *Hepeviridae*, also known as hepatitis E viruses (HEVs), infect a wide range of mammalian and avian species as well as trout. Hepeviruses which infect humans can cause self-limiting acute hepatitis in immune competent individuals (Smith et al., 2014). All mammalian and avian HEV isolates belong to the genus *Orthohepevirus*, and can be divided into four species. The species *Orthohepevirus A* consists of HEV variants detected in human populations worldwide and those from pigs, wild boars, camels, some of which can be transmitted to humans and cause zoonotic diseases. The species *Orthohepevirus C* is composed of two genotypes with host restriction. All HEVs identified from rodent hosts are designated genotype C1, while genotype C2 includes isolates derived from ferrets (Smith et al., 2014). We discovered a novel HEV within genotype C1 from a *R. rattus* in Kenya, further suggesting the global distribution of *Orthohepevirus C* variants. Previous studies revealed that HEVs detected in Asian musk shrew (*Suncus murinus*) from different regions of China were closely related to rat HEVs and fell within HEV-C1 genotype. As those shrews always share the same environment with wild rats, they may carry rat HEVs as a result of the cross species transmission from the wild rat reservoirs (Guan et al., 2013; He et al., 2018). In this study, we detected a distinct HEV in *C. olivieri*, a shrew species native in Africa, which is phylogenetically distant from rat HEVs. Full-length genome sequence of the new shrew HEV needs to be obtained to judge whether it can be classified as a novel species or it remains within *Orthohepevirus C* as a new genotype, following the demarcation criteria for *Orthohepevirus* species by the ICTV. In order to better understand the evolutionary position of shrew HEVs in Hepeviridae family, virus sequences from a greater diversity of shrew host species and geographic origin need to be determined. The zoonotic potential of rodent and shrew HEVs is also yet to be characterized.

The genus *Mammarenavirus* consists of two main groups corresponding to Old-World and New-World mammarenaviruses, which are the causative agents of a variety of human hemorrhagic fever diseases in Africa and America, respectively (Radoshitzky et al., 2015). Rodents are the primary natural reservoir of mammarenaviruses and have been the major target wildlife for surveillance of this category of viruses in nature. Among Old-World mammarenaviruses, besides the worldwide spread Lymphocytic choriomeningitis virus (LCMV) and Wenzhou-related viruses which have recently been widely reported in China and southeast Asia (Wang et al., 2019), all other viruses have been exclusively found in Africa, with each virus hosted by a specific host species or group of species (Charrel and de Lamballerie, 2010). These include LASV, Gbagroube virus, and Menekre viruses in West Africa; Ippy and Luna viruses in Central Africa; Morogoro, Gairo, and Mobala viruses in East Africa; Mopeia, Mariental, and Merino walk virus (MWV) in Southern Africa (Ishii et al., 2012; Gryseels et al., 2015). In Kenya, regardless of its large rodent diversity and the prevalent populations of *M. natalensis*, one of the most important host species of arenavirus, there are no records of arenavirus in rodents up to date. In this study, we characterized an arenavirus from rodents in Kenya for the first time. The novel virus from *G. macmillani* termed Kitale virus shows significant sequence distinction from all previously known arenaviruses in both of the two genomic segments, as well as in the four ORFs. Our results not only report the first arenavirus detection in Kenya, but also present the discovery of a new mammarenavirus species from a novel species of rodent host.

A number of OWA are associated with human diseases. LASV causes Lassa hemorrhagic fever with 5,000–10,000 cases of death per year in West Africa (Fichet-Calvet and Rogers, 2009). Another pathogenic arenavirus in Africa, Lujo virus, was discovered in South Africa following a nosocomial infection occasioned by a patient who traveled from Zambia (Ishii et al., 2012). In Asia, a genetic variant of Wenzhou virus was reported to have been associated with influenza-like human respiratory illness in Cambodia (Blasdell et al., 2016). The pathogenicity of other OWA remains unclear. However, Ippy virus, to which Kitale virus is most closely related, was found to be antigenically a member of the Lassa fever complex of arenaviruses via cross-immunofluorescence test (Swanepoel et al., 1985). While the pathogenic potential of Kitale virus requires further studies, the spillover risk of this novel rodent arenavirus should not be neglected. Additionally, the detection of Kitale virus in all tested tissues of the infected animal suggests its wide tissue tropism in the natural host. This may increase the potential of being transmitted to humans by enabling viral shedding into the environment through numerous routes such as saliva, urine, and feces.

As increased disturbance of natural habitats has brought rodents and shrews in closer contact with people, these small mammals are likely to be the next source of zoonotic disease outbreak in Kenya. Therefore, identification of known and novel viruses in rodents and shrews is of public health significance. In this study, diverse novel RNA viruses of different families were discovered in rodents and shrews from regions of intensive agricultural activities, including a novel arenavirus related to those known to infect humans. The findings extend our knowledge on the host range, ecological distribution, and evolution of these RNA viral families. It also highlights the need for continued pathogen surveillance and disease monitoring not only in risk wildlife but also in livestock and humans in Kenya to prevent the occurrence of future zoonotic diseases.

## Data Availability Statement

All sequences generated by this study were deposited in the GenBank under accession numbers MK935152–MK9345169.

## Ethics Statement

The study was approved by the Research and Ethics Committee of the Kenya Wildlife Service, with research permit KWS/BRM/5001. All Institutional and National guidelines for care and handling use of animals were followed.

## Author Contributions

Z-LS, BA, BH, and SOO conceptualized and designed the study. Z-LS, BA, BH, SO, and VO coordinated the study and the field work. BA, SO, VO, and SOO participated in the field work. Z-LS and BA supervised the study. BA, SOO, BH, and X-LY designed and coordinated the experiments. SOO and GO managed the storage and retrieval of specimen. BL and BH coordinated the laboratory skills training. BA and VO were responsible for application and acquisition of ethics permit. SOO performed the experiments, and analyzed and interpreted the data. KK, YF, and X-SZ assisted with data analysis. SOO and BH drafted the manuscript.

## Conflict of Interest

The authors declare that the research was conducted in the absence of any commercial or financial relationships that could be construed as a potential conflict of interest.
